# Hypocalcemic tetany associated with simultaneous administration of cimetidine and nifedipine: a case report

**DOI:** 10.1186/s13256-023-03811-6

**Published:** 2023-07-28

**Authors:** Yihienew M. Bezabih, Mekides A. Bimrew, Woldesellassie M. Bezabhe

**Affiliations:** 1 Shedeho-Meket Primary Hospital, North Wollo, Ethiopia; 2 Arsi University College of Health Sciences, Arsi University, P. O. Box, 394, Arsi, Ethiopia; 3 Hobart, TAS 7007 Australia; 4 School of Pharmacy and Pharmacology, University of Tasmania, Private Bag 26, Hobart, TAS 7001 Australia

**Keywords:** Hypocalcemic tetany, Carpopedal spasm, Cimetidine, Nifedipine, Case report

## Abstract

**Background:**

Acute hypocalcemia is generally caused by a sudden drop in serum calcium ion and presents with a mild or severe form of tetany. Even though the occurrence of hypocalcemia is well documented with certain drugs such as calcium chelators, bisphosphonates, and cisplatin, it is a very unusual and poorly documented adverse event with cimetidine and nifedipine. Here, we present a case of severe hypocalcemic tetany during simultaneous administration of cimetidine and nifedipine in a hypertensive patient with dyspepsia.

**Case presentation:**

A 46-year-old known human immunodeficiency virus patient from Ethiopia on antiretroviral therapy over the past 14 years presented to the emergency department with acute exacerbation of dyspepsia and hypertensive urgency. She was given intravenous cimetidine (400 mg) and oral nifedipine (30 mg) simultaneously. One hour after the administration of these two drugs, she developed severe hypocalcemic tetany with carpopedal spasm, involuntary plantar flexion, and muscle spasms. She also had severe retrosternal chest pain and shortness of breath. Her blood pressure was 160/110 mmHg during the attack and she had no skin changes, such as urticaria. She was immediately given 1 g of calcium gluconate intravenously over 30 minutes. The carpopedal spasm progressively decreased during calcium gluconate administration. An hour later, she completely regained voluntary movement of her fingers and feet. The chest pain persisted, but resolved over the next 12 hours. The patient was discharged home after 2 days of observation. This is an unusual adverse effect that needs caution during concomitant administration of these drugs.

**Conclusions:**

Severe hypocalcemic tetany can occur with concomitant administration of cimetidine and nifedipine. Immediate treatment with calcium gluconate quickly reverses this adverse event. Concomitant administration of these drugs should be done with caution or be avoided if possible.

## Background

Hypocalcemia commonly results from disorders of parathyroid hormone (PTH) and vitamin D [1]. It also occurs whenever other factors that influence serum calcium are deranged. These include factors that increase the binding of free serum calcium ion to albumin (for example, alkalosis) or conditions that cause precipitation of serum calcium (hyperphosphatemia, citrated blood transfusion, and certain drugs such as foscarnet) [1]. While a defect in calcium homeostasis gives subacute to chronic hypocalcemia, acute hypocalcemia is generally caused by a sudden drop in serum calcium ion due to its precipitation or a shift from the circulation [1]. A typical characteristic feature of acute hypocalcemia involves neuromuscular irritability resulting in tetany. The symptoms of tetany may be mild (peri-oral numbness, paresthesias of the hands and feet, muscle cramps) or severe (carpopedal spasm, laryngospasm, and focal or generalized seizures) [1].

Even though hypocalcemia following the administration of certain drugs including calcium chelators, bisphosphonates, cinacalcet, and cisplatin is well documented, hypocalcemic tetany with cimetidine and/or nifedipine is very unusual, mechanistically unexplainable, and a poorly documented adverse event. Here, we present a case of severe and life threatening hypocalcemic tetany in a patient with human immunodeficiency virus/acquired immunodeficiency syndrome (HIV/AIDS) who was given oral nifedipine and intravenous cimetidine.

## Case presentation

A 46-year-old known HIV patient from Ethiopia who was receiving antiretroviral therapy (ART) for the past 14 years and was a known hypertensive on dietary management, presented to the emergency department with epigastric pain that radiates to the back. She was taking triple ART therapy [tenofovir (TDF), lamivudine (3TC), and dolutegravir (DTG)] and had good self-reported medication adherence. She came to the emergency room walking by herself. Her vital signs at arrival were blood pressure (BP) 210/130 mmHg, pulse rate (PR) 96 beats per minute (bpm) regular and full in volume, respiratory rate (RR) 20 breaths/minute, and temperature (T°) 37.1 °C. She had no abdominal tenderness and physical examination findings in other systems were also nonremarkable.

She was given cimetidine 400 mg intravenous stat and then nifedipine 30 per os (P.O) stat 5 minutes later. One hour after administration of these two drugs she developed carpopedal spasm involving the fingers and was unable to stand due to spasm with rigid plantar flexion. She also developed severe retrosternal chest pain that became extremely severe, shivering, chills, and shortness of breath. There was no rash or any skin reaction and her BP was 160/110 mmHg. She was immediately put on intranasal oxygen and was given morphine 5 mg intravenous stat and calcium gluconate 1 g intravenous over 30 minutes. The carpopedal spasm progressively decreased during calcium gluconate administration and she completely regained voluntary movement of her fingers and foot an hour later. However, the retrosternal chest pain did not improve and she was given additional doses of morphine and an electrocardiogram (ECG) did not show any sign of myocardial ischemia. Twelve hours later, the retrosternal pain decreased and she became stable sitting on her bed with all symptoms improved except the mild epigastric discomfort. Repeat ECG showed no sign of ischemia or infarction.

## Investigations

Her total calcium level was 7.1 mg/dL from the blood sample drawn before calcium gluconate was administered, and 8.8 mg/dL 2 days later. Random blood glucose was 116 mg/dL during the tetany. Serum albumin was within normal range (5 g/L). Other serum electrolytes (Na^+^  140.7 mmol/L, K^+^ 4.2 mmol/L, Cl^−^ 102.3 mmol/L) were within the normal range. Her CD4 count was 573 and her complete blood count (white blood cell count 4.4 × 10^3^ with neutrophil 56% and lymphocyte 28%, hemoglobin 13.3 g/dL), renal function (creatinine 0.7 mg/dL, urea 18 mg/dL) and liver function [aspartate aminotransferase (AST) 43 U/L, alanine aminotransferase (ALT) 48 U/L, alkaline phosphatase (ALP) 232 U/L) tests were all within the normal limits. Brain computerized tomography (CT)was also performed and there was no evidence of any intracranial lesion. Laboratory tests for free calcium ion, serum PTH, vitamin D, and phosphate were not performed as these tests were not available at the hospital.

## Differential diagnosis

This patient had a classical severe hypocalcemic tetany that responded to the administration of intravenous calcium gluconate. The tetany also had temporal association with administration of the responsible drugs. However, since the patient was a known HIV patient that came with a hypertensive urgency, seizure due to an intracranial space occupying lesion or a stroke was a top differential diagnosis. This was, however, ruled out with a normal brain CT scan. Other possible differentials included sepsis, panic attack with hyperventilation, and intake of drugs known to cause hypocalcemia. However, she had no features of serious infection (no fever, tachycardia, or leukocytosis) had no hyperventilation before the onset of tetany. She was not on any form of chemotherapy and the only medications she was taking were the antiretrovirals. Her antiretroviral drugs (TDF, 3TC, and DTG) were checked for any interactions and they did not have a calcium lowering effect when taken with either cimetidine or nifedipine [2]. She had no history of surgery around the neck. In addition, HIV patients are prone to vitamin D deficiency related to malnutrition and malabsorption [3]. Such homeostatic abnormalities, however, would rather have more chronic than acute presentation.

## Treatment, outcome, and follow-up

Following the hypocalcemic tetany, nifedipine and cimetidine were immediate discontinued. We then used intravenous omeprazole to treat her acute exacerbation of dyspepsia and enalapril and hydrochlorothiazide (HCT) for the hypertension. Her BP was stabilized with these two oral medications (enalapril 5 mg P.O BID and HCT 25 mg P.O daily). After 2 days of stay in emergency department, she was discharged home with an appointment after 1 week. In this follow-up visit, the patient was in a stable condition without any symptoms and her BP was 150/90 mmHg. She was ambulating without any difficulty and the Chvostek and trousseau signs were negative. We gave her vitamin D_3_ (1000 IU/day) and she was advised on low salt diet and the need to continue her antihypertensive and ART medications. We also gave the patient a written documentation to avoid simultaneous intake of cimetidine and nifedipine in the future.

## Discussion and conclusions

This case-report described a case of severe symptomatic hypocalcemia after intravenous administration of cimetidine and oral nifedipine.

There are few reports of hypocalcemia after cimetidine administration [4–6] even though the mechanisms are less clear. Edwards *et al*. [4] described a case of hypocalcemic tetany in a 92-year-old woman who received prophylactic cimetidine (400 mg QID) following sigmoid resection. This woman had normal serum calcium prior to cimetidine administration and developed low serum calcium, tetany, seizures, and impaired mental status after receiving cimetidine. The patient’s symptoms showed rapid suppression with gluconate calcium treatment. A study by Sherwood *et al*. [6] showed an interesting effect of cimetidine (300 mg P.O QID) in 12 hypercalcemic patients due to primary hyperparathyroidism. In all 12 patients, cimetidine treatment decreased serum calcium and parathyroid hormone levels. Interestingly, when cimetidine was discontinued, serum calcium and PTH levels began to rise within few hours. This suggests that cimetidine might decrease the synthesis/release of PTH hormone [4, 6]. Another double-blind, controlled trial was undertaken to prove the use of cimetidine for possible treatment of primary hyperparathyroidism involving 16 patients that received either oral or intravenous cimetidine. One patient (out of 16 patients) developed a significant fall in serum calcium with cimetidine intake.

Studies on the effects of nifedipine on parathyroid hormone function were conflicting. Some studies reported a decreased serum PTH level [7, 8], while others reported a rather increase in PTH [9, 10] after use of calcium channel blockers. One study reported hypocalcemic tetany in a pregnant patient who received nifedipine and intravenous magnesium sulfate. In this report, authors believed that magnesium-related hypocalcemia became symptomatic due to co-administration of nifedipine. A prospective study on 11 postmenopausal women taking nifedipine 30 mg P. O daily found a marked decrease in PTH hormone after 1 month of treatment [7]. In this study the ratio of serum calcium (after/before nifedipine) was < 1 in six patients, > 1 in four patients, and 1 in one patient.

The limitations of this case report were the lack of some laboratory investigations, including free calcium ion, serum PTH, vitamin D, and phosphate. Hence, there is a possibility that the patient might have undiagnosed vitamin D deficiency. Therefore, it is likely that the patient might have had asymptomatic hypocalcemia prior to presentation and the medications administered at the hospital worsened it. The strength of this report is that we ruled out other likely differential diagnoses.

This is the first report of severe hypocalcemic tetany due to the synergistic effects of cimetidine and nifedipine. The synergistic effect could arise from the possible tendency of these drugs to cause hypocalcemia. In addition, when co-administered, cimetidine might increase the concentration of nifedipine through inhibition of oxidative liver metabolism [2]. Although hypocalcemic tetany is an uncommon adverse effect, it should be noted that simultaneous administration of cimetidine and nifedipine can lead to life-threatening hypocalcemia. We recommend that caution should be taken with concomitant administration of these drugs.

## Data Availability

All data generated or analysed during this study are included in this published article.

## Abbreviations

3TC: Lamivudine
ALP: Alkaline phosphatase
ALT: Alanine aminotransferase
ART: Antiretroviral therapy
AST: Aspartate aminotransferase
BID: Twice a day
BP: Blood pressure
CD4 count: Number of T cells bearing CD4 on their surface
CT: Computerized tomography
DTG: Dolutegravir
ECG: Electrocardiogram
g/L: Gram per liter
HIV: Human immunodeficiency virus
HCT: Hydrochlorothiazide
IV: Intravenous
IU: International unit
mg: Milligram
mg/dL: Milligram per deciliter
mmHg: Millimeter of mercury
mmol/L: Millimoles per liter
P.O: Per os (by mouth)
PTH: Parathyroid hormone
PR: Pulse rate
QID: Four times a day
RR: Respiratory rate
T°: Temperature
TDF: Tenofovir
U/L: Units per liter
